# Small (autonomic) and large fiber neuropathy in Parkinson disease and parkinsonism

**DOI:** 10.1186/s12883-016-0667-3

**Published:** 2016-08-17

**Authors:** Davi Farias de Araújo, Antônio Pinto de Melo Neto, Ítalo Sérgio Cavalcante Oliveira, Beatriz Soares Brito, Ineusi Teixeira de Araújo, Ingrid Sousa Barros, José Wellington Oliveira Lima, Wagner Goes Horta, Francisco de Assis Aquino Gondim

**Affiliations:** 1 Federal University of Ceará (UFC), Professor Costa Mendes Street, 949, Fortaleza, Ceará Brazil; 2 Departamento de Saúde Comunitária, State University of Ceará (UECE), Fortaleza, Ceará Brazil; 3 Department of Internal Medicine, Neurology Division, Federal University of Ceará, Professor Costa Mendes Street, 1608, Fortaleza, Ceará Brazil

**Keywords:** Neuromuscular disorders, Parkinson’s disease, Parkinsonism, Skin Wrinkling test, Peripheral neuropathy

## Abstract

**Background:**

Recent studies have reported that peripheral neuropathy (PN) is common in patients with Parkinson’s disease (PD) and raised the possibility that levodopa neurotoxicity is the main culprit.

**Methods:**

We evaluated the presence of large & small (autonomic) fiber PN in 54 consecutive patients with PD or parkinsonism in a tertiary outpatient clinic from Brazil. Initial PN screening consisted of history/neurological exam and skin wrinkling test (SWT). In addition, we also performed Nerve conduction studies/Electromyography (NCS/EMG) in all patients with PN signs/symptoms and/or abnormal SWT.

**Results:**

Thirty eight patients with PD (10 women, mean age: 63 ± 2.1 years, *P* < 0.05 versus parkinsonism, mean disease duration: 8 ± 0.8 years) and 16 patients with other forms of parkinsonism [7 women, mean age: 50.1 ± 3.9 years, mean disease duration: 6.9 ± 1.1 years] completed clinical neuromuscular evaluation. SWT was performed in 48 patients (33 PD, 15 parkinsonism). In the PD group, SWT was abnormal in 57.6 % of the tested patients (comprising 50 % of all PD patients). In the parkinsonism group, SWT was abnormal in 37.5 % (comprising 35.3 % of all parkinsonism patients). NCS/EMG was performed in 39 patients (26 PD and 13 parkinsonism). Twelve out of the 26 PD (34.2 % of all PD) and 4 out of the 13 parkinsonism (23.5 % of all parkinsonism) had abnormal NCS/EMG results. Neuropathy prevalence was similar in PD and parkinsonism groups as detected either by NCS/EMG or SWT.

**Conclusions:**

Large fiber and small (autonomic) fiber PN are common in patients with PD and parkinsonism. The etiology for the neuropathy was likely to be multifactorial and may be secondary to PD itself.

**Electronic supplementary material:**

The online version of this article (doi:10.1186/s12883-016-0667-3) contains supplementary material, which is available to authorized users.

## Background

Although autonomic involvement was extensively described in Parkinson’s disease (PD) and other forms of parkinsonism (e.g. multiple system atrophy, Lewy body disease), the presence of sensorimotor peripheral neuropathy (PN) was only detailed in case reports and small case series until recently [1, 2]. In the early 2000s, peripheral neuropathy was thought to be present in genetic forms of Parkinson’s disease [3, 4]. In 2008, Toth et al were among the first to report that a significant percentage of patients with PD were affected by peripheral neuropathy [5]. Toth et al. reported symptomatic neuropathy in 9.8 % of 500 PD patients, and attributed that to high homocysteine levels due to levodopa use (levodopa effect on vitamin B12 metabolism) [5]. The same group also subsequently reported higher neuropathy prevalence (about 6x greater than controls) in a case-control study of PD patients [6]. Neuropathy was axonal, more prevalent in older patients with high homocysteine/methylmalonic acid levels and higher Unified Parkinson’s disease rating scale (UPDRS) scores. A multicenter study in Italy also reported prevalence of 19.4 % of neuropathy in PD patients, due to long term levodopa exposure, older age and high homocysteine/methylmalonic acid and low vitamin B12 levels [7].

In 2005, we started a study to screen neuropathy in Brazilian patients with PD. Although full neuropathy screening could not be initially completed due to financial constraints, we reported preliminary findings of 10 symptomatic PD patients with neuropathy [8]. In our study, neuropathy phenotype was diverse and included demyelinating neuropathies. Co-morbidities were also different from previous studies and included monoclonal gammopathy of uncertain significance and hypothyroidism [8]. Recently, additional observations have broadened the clinical spectrum of peripheral neuropathies in PD patients to include demyelinating and small fiber neuropathies [9–11]. Here, we describe the results of a study aimed to screen large and small (autonomic) fiber neuropathy in patients with PD and parkinsonism. Screening for small (autonomic) fiber neuropathy was done by skin wrinkling test, an inexpensive bedside diagnostic tool that was recently considered to be comparable to skin biopsy testing. Part of this study has been reported in abstract form elsewhere [12].

## Methods

### Subjects

All consecutive patients with the diagnosis of Parkinson’s disease or parkinsonism (secondary to Willson disease *N* = 4, progressive supranuclear palsy/corticobasal degeneration *N* = 3, multiple system atrophy *N* = 3, Lewy body disease *N* = 1, vascular parkinsonism *N* = 1 and unestablished etiology *N* = 4) seen at a newly established tertiary outpatient clinic at the Federal University of Ceará were offered to participate in this study. Patients were enrolled over a period of 1 year and one additional year was necessary to complete the diagnostic testing and laboratory work-up. Very few patients declined to participate in the study (the authors estimate that less than 10 % of the eligible patients) and no patient withdrew from the study after initial enrollment.

### Techniques and experimental design

The study consisted in a standard evaluation for the presence of large and small (autonomic) fiber peripheral neuropathy. Fifty-four patients were enrolled in approximately 1 year and completed the neuromuscular evaluation. A board-certified neurologist (by both the American Academy of Neurology and Academia Brasileira de Neurologia) conducted the initial PN screening. It consisted of history gathering (e.g. complaints of paresthesias, burning feet, unexplained worsening gait) and detailed neurological exam looking for classicial findings of neuropathy (predominantly distal atrophy, decreased or absent deep tendon reflexes, length-dependent sensory loss). Thereafter, a group of medical students supervised by the corresponding author conducted the skin wrinkling test (SWT), according to the same protocol described by Teoh et al [13] that we had also previously employed [14]. Briefly, the SWT consisted of hand immersion in NaCl at 0.5 mmol/l at 40.5^o^C during 30 min. Thereafter, skin wrinkling was visually graded in 4 digits (all but the thumb) and a mean score of wrinkling was created. Patients were considered to have neuropathy if mean SWT score was lower than 2. If a patient had signs or symptoms of peripheral neuropathy and/or an abnormal SWT, this initial evaluation was followed by a nerve conduction study/electromyography (NCS/EMG). The final result of the neuromuscular evaluation was based on the combined results of the neurological exam, SWT and NCS/EMG. For the lower extremities, the following cut-offs were used to diagnose abnormalities in the nerve conduction studies: peroneal compound muscle action potential (CMAP) amplitudes (2.5 mV), tibial CMAP amplitudes (3.5 mV), sural sensory nerve action potential (SNAP) amplitudes (4 uV) and tibial, peroneal and sural nerve conduction velocities (39 m/s). Absent sural responses were not considered to be necessarily abnormal in patients older than 60 years.

The medical charts of all patients were also reviewed, including the risk factors for neuropathy. Basic laboratory tests were requested to pursue the work-up for neuropathy and/or clinical evaluation for the presence of thyroid diseases, diabetes mellitus (DM) or vitamin B12 deficiency. Although most of the patients routinely had prior evaluation for diabetes and thyroid disease by primary care physicians (although not all test results were available), screening for B12 deficiency could only be documented in 63.2 % of the PD and 56.3 % of the parkinsonism group.

### Statistics and ethics

This study was approved by the local Institutional Review Board of the Universidade Federal do Ceará (Comitê de Ética em Pesquisa do Hospital Universitário Walter Cantídio). All patients signed an informed consent prior to the enrollment in this study. Descriptive statistics followed by Chi-square and Fischer exact test and parametric (*t* test) and non-parametric tests (Mann-Whitney test) were then performed (choice according to Gaussian or non-Gaussian distribution). Thereafter, univariate regression analysis was performed to establish whether each variable was a risk factor for neuropathy. One-Way ANOVA followed by the Holm-Sidak test was used to compare the results of the electrodiagnostic tests. Results are detailed as mean ± SEM and differences were considered significant if *P* < 0.05.

## Results

### Clinical and demographic features

Table 1 compares the demographic characteristics of the 38 patients with PD and 16 patients with other forms of parkinsonism that completed our work-up. Patients with PD were older than patients with parkinsonism (*P* < 0.05, Mann Whitney test). However, age of onset, disease duration and gender distribution were similar in both groups, despite a trend for higher percentage of women in the parkinsonism group and for older age of onset and longer disease course in the PD group. Mean Hoehn and Yahr scores in the PD group were 2.6 ± 0.1 (score of 2 indicates bilateral involvement with preservation of balance, while score of 3 means bilateral involvement with mild-moderate impairment of postural reflexes). Most of the PD patients were treated with levodopa and 68.8 % of the parkinsonism group were taking levodopa. A third of the PD patients and 31 % of the parkinsonism group were treated with pramipexol while 38.9 % of the PD and none from the parkinsonism group were treated with amantadine and only 8.3 and 18.8 (respectively) were treated with biperiden.

**Table 1:** Demographic characteristics and risk factors for neuropathy in patients with Parkinson’s disease and Parkinsonism

	Parkinson’s disease (*N* = 38)	Parkinsonism (*N* = 16)
Age	63 ± 2.1	50.1 ± 3.9*
% Female	26.3	43.8
Age of onset (years)	54.3 ± 2	42.1 ± 4.3
Disease duration (years)	8 ± 0.8	6.9 ± 1.1
Definite and Possible Risk Factors for Neuropathy		
% Diabetes mellitus	10.5	25
% Thyroid disease	6.6	0
% B12 level <200	13	22.2
% B12 level <300	20.8	44.4
% Levodopa use	97.2	68.8
Neurophysiological studies		
Peroneal CMAP (mV)	3.99 ± 0.47	3.8 ± 0.66
Peroneal CV (m/s)	46.9 ± 1.02	49.3 ± 1.56
SNAP (μV)	11.9 ± 1.16	14.5 ± 3.77
Sural CV (m/s)	48.9 ± 0.88	46.3 ± 1.12
Tibial CMAP (mV)	8.67 ± 0.88	9.8 ± 1.3
Tibial CV (m/s)	47 ± 0.76	46.2 ± 1

*P < 0.05, Student *t* test

### Results of small (autonomic) fiber neuropathy assessment

Skin Wrinkling Test (SWT) was performed in 49 patients, 33 with PD and 15 with other forms of parkinsonism. No statistical difference was observed in the percentage of patients with abnormal SWT between the 2 groups: 19 out of 33 of the PD group (57.6 %, mean SWT grade: 1.98 ± 0.2) and 6 out of 15 of the patients with parkinsonism (40 %, mean SWT grade: 2.3 ± 0.3). Among the patients with evidence of small (autonomic) fiber neuropathy in the PD group, 10 % had diabetes, 5 % thyroid disease, 5 % B12 deficiency and 5 % had been treated for leprosy in the past while additional 15 % had B12 levels <300. Among the patients with evidence of small fiber neuropathy in the parkinsonism group, 12.5 % had diabetes mellitus. Therefore, in 65 % of the PD patients and among 87.5 % of the patients with parkinsonism, no common etiology of peripheral neuropathy was identified.

### Results of large fiber neuropathy assessment

NCS/EMG was performed in 39 patients (26 PD and 13 with other forms of parkinsonism). 12 out of the 26 PD (34.2 % of all PD) and 4 out of the 13 from the parkinsonism group (23.5 % of all patients from the parkinsonism group) had abnormal NCS/EMG results. As expected, patients with abnormalities suggestive of peripheral neuropathy on the screening neurological exam were more likely to have abnormal EMG (*P* = 0.04, Fischer exact test). Neuropathy prevalence was similar in the groups with PD and parkinsonism (*P* > 0.05), whether PN was assessed by SWT, NCS/EMG or clinically (final combined neuromuscular evaluation).

**Fig. 1:**
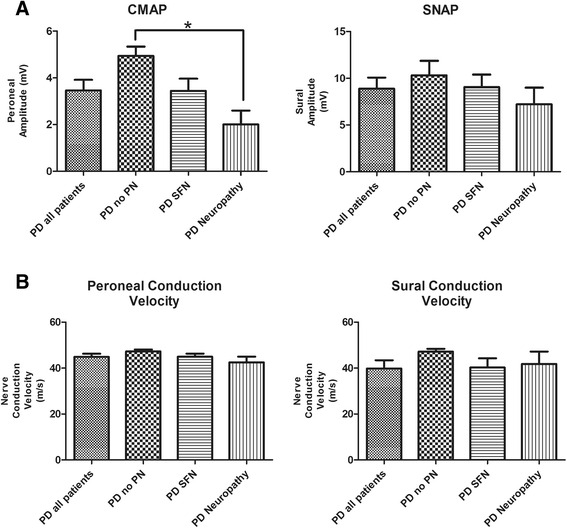
Part **a** shows mean peroneal compound motor action potential (CMAP, in mV) amplitudes and mean sural sensory nerve action potential (SNAP, in μV) amplitudes in patients with Parkinson’s disease (PD). “PD all patients” refers to mean values from the whole group (all patients with PD), “PD no PN” refers to mean values from PD patients without large fiber neuropathy, “PD SFN” refers to patients with PD and small fiber neuropathy and “PD Neuropathy” to mean values in patients with PD and large-fiber neuropathy. Part **b** shows mean values of peroneal motor and sural sensory conduction velocities in the same groups

**Fig. 2:**
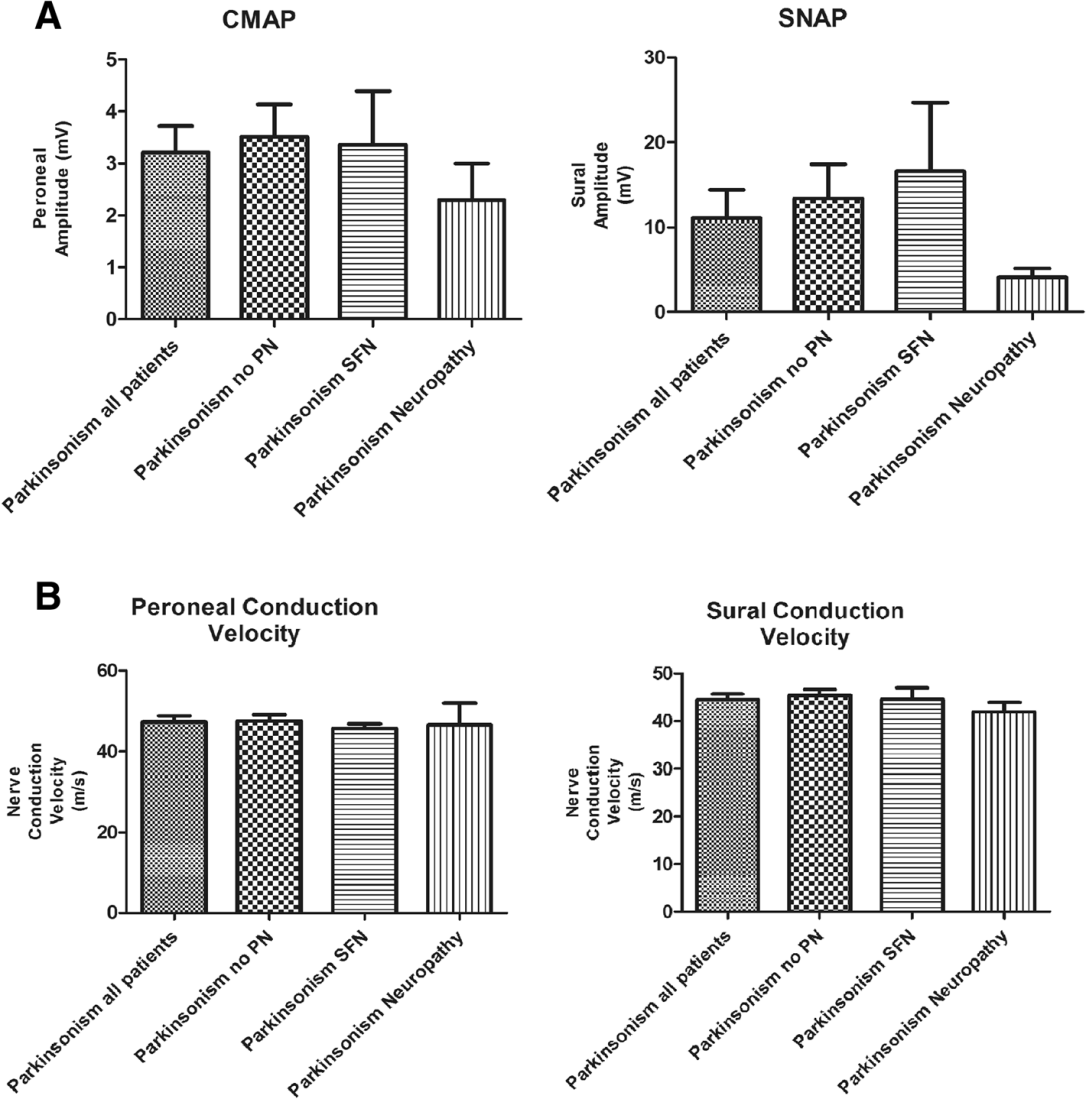
Part **a** shows mean peroneal compound motor action potential (CMAP, in mV) amplitudes and mean sural sensory nerve action potential (SNAP, in μV) amplitudes in patients with parkinsonism. “Parkinsonism all patients” refers to mean values from the whole group (all patients with parkinsonism), “Parkinsonism no PN” refers to mean values from parkinsonism patients without large fiber neuropathy, “Parkinsonism SFN” refers to patients with parkinsonism and small fiber neuropathy and “Parkinsonism Neuropathy” to mean values in patients with Parkinsonism and large-fiber neuropathy. Part **b** shows mean values of peroneal motor and sural sensory conduction velocities in the same groups

Table 1 displays the results of the most important neurophysiological parameters from the lower extremities nerve conduction studies in patients with PD and other forms of parkinsonism. No statistically significant difference was evidenced between PD and other forms of parkinsonism for the tibial and peroneal motor amplitudes and conduction velocities as well as from sural sensory amplitudes and conduction velocities. Figures 1 and 2 further detail the electrodiagnostic findings in patients with PD and parkinsonism with and without large and small fiber neuropathy. As can be seen in Fig. 1a, patients with large-fiber neuropathy and PD had lower peroneal CMAP amplitudes than patients without large-fiber neuropathy (*P* < 0.01). Figures 1a and 2a also reveal that despite a trend for lower sural SNAP amplitudes in patients with PD and large-fiber neuropathy, there was no statistically significant difference in sural SNAP amplitudes in patients with PD or parkinsonism with and without large-fiber neuropathy. Also, as can be seen in Figs. 1b and 2b, peroneal and sural conduction velocities in patients with PD or parkinsonism were not significantly different in patients with small or large-fiber neuropathy.

### Risk factor assessment for large and small (autonomic) fiber neuropathy

Patients with EMG abnormalities were older: 65.5 ± 3.8 vs. 53 ± 3 years (*P* = 0.01, Mann-Whitney test), but abnormalities on SWT scores were not associated with age. In addition, patients with either SWT or EMG abnormalities were not more likely to be older than normal patients. Patients with EMG abnormalities were more likely to be older at the disease onset (57.2 ± 3.8 versus 45.3 ± 3.1 years, *P* = 0.01) but there was no significant relationship between EMG abnormalities and disease duration. In addition, there wasn’t any significant relationship between the age of disease onset or disease duration and SWT scores (*P* = 0.44).

Univariate regression analysis showed a significant correlation between age and EMG abnormalities (*P* = 0.01) but not with SWT scores (*P* = 0.53). Univariate regression analysis also showed a significant correlation between age of onset and EMG abnormalities (*P* = 0.03). Patients with higher Hoehn and Yahr stage scores were more likely to have EMG abnormalities (Fischer exact test, *P* = 0.02) than patients with lower scores, but no significant difference was found between SWT and Hoehn and Yahr stage scores. Patients taking biperiden were more likely to have EMG abnormalities (*P* = 0.05, Fischer’s exact test). Patients with neuropathy were not more likely to have diabetes mellitus, hypothyroidism, B12 deficiency, use levodopa, pramipexole or amantadine. Almost all patients from the PD group were taking levodopa but patients with PD were more likely to take levodopa than patients with parkinsonism (*P* = 0.003). However, the total amount of levodopa exposure on each patient was not quantified, and therefore, the cumulative effect of levodopa on the neuropathy development could not be assessed.

Regression analysis of tibial and peroneal CMAP amplitudes and conduction velocities or sural SNAP and conduction velocities versus the presence of DM or B12 levels was not significant. In addition, regression analysis also did not disclose any significant relationship between SWT scores and electrodiagnostic parameters, i.e. tibial and peroneal CMAP amplitudes and conduction velocities or between sural SNAP and conduction velocities.

## Discussion

Our study revealed that large fiber and small (autonomic) fiber PN are common in patients with PD and other forms of parkinsonism. Those findings are consistent with several prior studies that demonstrated that large fiber neuropathy is highly prevalent in PD patients [6–9]. A recent review evaluated all published studies about this topic [15].

However, to our knowledge, this is the first study to compare the prevalence of neuropathy in patients with PD and parkinsonism and where a systematic screening for small (autonomic) fiber neuropathy (clinical and with skin wrinkling test) was conducted in both groups (patients with PD and other forms of parkinsonism). We observed that 57.6 % of the PD group (50 % of all PD) and 37.5 % of the patients with parkinsonism (35.3 % of all patients from the parkinsonism group) who underwent SWT had an abnormal SWT result. These findings are consistent with several recent studies that attempted to evaluate the involvement of the small fibers in patients with PD. Nolano et al. were the first to report significant reduction of epidermal nerve fibers in 100 % of the PD patients [16]. Wang et al. have described deposits of α-synuclein in autonomic but not in sensory nerve fibers in 100 % of the PD patients [11]. Higher α-synuclein ratios correlated with autonomic dysfunction and Hoehn and Yahr scores [11]. Donadio et al. have also reported anti-phosphorylated α-synuclein deposits in 100 % of the distal and proximal skin from PD patients [12].

One of the limitations of our small fiber neuropathy assessment is that we only employed SWT. We were not able to run a complete battery of autonomic tests, questionnaires for evaluation of autonomic dysfunction (e.g. COMPASS) or additional tests to evaluate sensory C fibers, such as skin biopsies. Skin wrinkling test (SWT) is an unexpensive and easy tool to detect abnormalities in small fiber function. SWT has been increasingly employed for small fiber evaluation and has demonstrated to be as accurate as skin biopsies for the diagnosis of small fiber neuropathy [13]. However, SWT more accurately evaluate the function of autonomic fibers and therefore not necessarily somatic small fibers. Among 65 % of the PD patients with small fiber neuropathy, no common etiology of peripheral neuropathy was identified. Exposure to levodopa was not a risk factor *per se* although we have not quantified the extent of levodopa exposure nor correlated the total exposure with the development of small fiber neuropathy. It is also important to emphasize that even when a possible etiology was identified, this abnormality cannot be necessarily attributed as the cause of neuropathy, since it may be a contributory and not the etiological factor.

Large-fiber neuropathy was not as prevalent as small fiber neuropathy in patients with either PD or parkinsonism due to other causes. As expected, they were more likely to have abnormalities suggestive of peripheral neuropathy on the neurological exam (*P* = 0.04). Patients with large-fiber neuropathy and PD had lower peroneal CMAP amplitudes than patients without large-fiber neuropathy (*P* < 0.01), but despite a trend for lower sural SNAP amplitudes in patients with PD and large-fiber neuropathy, sural SNAP amplitudes were not significantly different (Fig. 1a). This may be explained by the fact that patients with small fiber neuropathy may have earlier signs of sensory axonal involvement, therefore dropping the amplitudes of sural SNAP amplitudes and preventing them to reach the level of statistical significance. Peroneal (motor) and sural (sensory) conduction velocities were also not statistically different, reinforcing the presence of an axonal phenotype (no patient from the PD or parkinsonism group had demyelinating neuropathy). In most studies of neuropathy and PD, the sensorimotor axonal phenotype is predominant [5, 7, 15].

Although both large and small fiber neuropathy were highly prevalent in PD and parkinsonism patients, there was no correlation between SWT scores and electrodiagnostic parameters diagnostic of large-fiber neuropathy. In addition, patients with EMG abnormalities were older and had higher Hoehn and Yahr stage scores while abnormalities on SWT scores were not associated with age. Patients with EMG abnormalities (but not with SWT abnormalities) were more likely to be older at the disease onset but EMG abnormalities were not dependent on disease duration. Overall those findings suggest that small (autonomic) and large-fiber neuropathy have different profiles, with early small fiber involvement more linked to PD itself while large-fiber involvement may be secondary to a mixture of progression of small fiber disease related to age, disease severity, co-morbidities (such as diabetes and hypothyroidism) and possibly levodopa exposure. The contribution of the different comorbidities rather than true neuropathy determinants is further reinforced by the lack of correlation between tibial and peroneal CMAP amplitudes, conduction velocities or sural SNAP and conduction velocities versus the presence of DM or B12 as detected by regression analysis. It is also important to emphasize that we did not test for more controversial, but possible contributory factors, such as pyridoxine deficiency and it would useful to know the exact percentage of patients with large and small-fiber neuropathy detected by our screening tools in an age-matched population in the same institution.

## Conclusions

In summary, large fiber and small (autonomic) fiber peripheral neuropathies are common in patients with PD and parkinsonism. Neuropathy etiology seems to be multifactorial in PD patients, but possibly secondary to PD itself (especially small fiber involvement). Long-term follow-up of patients with the different contributory factors and evaluation of patients with early stage of PD will be important to further answer additional questions about the etiology of PN in patients with PD.

## Additional file

Additional file 1: Detailed information about specific diagnosis, demographic characteristics, neurophysiologic studies, drugs in use and risk factors for peripheral neuropathy of PD and Parkinsonism groups. (XLSX 16 kb)

## Abbreviations

CMAP: Compound muscle action potential
DM: Diabetes mellitus
EMG: Electromyography
NCS: Nerve conduction studies
PD: Parkinson’s disease
PN: Peripheral neuropathy
SFN: Small fiber neuropathy
SNAP: Sensory nerve action potential
SWT: Skin winkling test
